# Supplementary material for: The role of awareness of repetition during the development of automaticity in a dot-counting task
Source: PeerJ. 2018 Jan 31;6:e4329. doi: 10.7717/peerj.4329 (PMC5797452; doi:10.7717/peerj.4329)
Supplement: Supplemental Information 1 [file peerj-06-4329-s001.docx]

RT data

| group | block1 | block2 | block3 | block4 | block5 |
| --- | --- | --- | --- | --- | --- |
| 1 | 1670.04 | 1454.35 | 1410.83 | 1274.66 | 1172.77 |
| 1 | 2165.64 | 1836.35 | 1590.66 | 1356.43 | 1071.01 |
| 1 | 3127.63 | 2036.3 | 1512.44 | 1408.43 | 1450.23 |
| 1 | 2026.14 | 1736.05 | 1500.28 | 1107.8 | 1086.77 |
| 1 | 3339.98 | 3156.85 | 2797.8 | 2638.64 | 2445.75 |
| 1 | 3905.33 | 3039.36 | 2796.1 | 2245.21 | 1721.97 |
| 1 | 4710.41 | 4328.61 | 3724.79 | 3706.16 | 3428.08 |
| 1 | 2921.96 | 2790.36 | 2806.98 | 2394.1 | 2305.11 |
| 1 | 3306.49 | 2658.53 | 2409.14 | 2303.85 | 2184.92 |
| 1 | 3206.69 | 2901.03 | 2688.14 | 2463.28 | 2023.25 |
| 1 | 2641.06 | 2346.18 | 1936.58 | 1491.98 | 1517.63 |
| 1 | 2465.94 | 2316.79 | 2101.86 | 1806.99 | 1649.35 |
| 1 | 2403.86 | 1633.06 | 1499.48 | 1412.34 | 1328.85 |
| 1 | 2524.31 | 2053.51 | 1990.47 | 1733.43 | 1779.78 |
| 1 | 2330.46 | 1795.09 | 1732.25 | 1627.96 | 1331.24 |
| 1 | 2869.93 | 2684.94 | 2268.32 | 1875.49 | 1608.85 |
| 1 | 2490.85 | 1683.33 | 1468.75 | 1234.38 | 1174.14 |
| 2 | 1793.88 | 1290.3 | 1064.91 | 945.24 | 888.53 |
| 2 | 2331.21 | 2095.04 | 2016.24 | 2070.17 | 1797.89 |
| 2 | 1779.75 | 947.61 | 823.37 | 739.07 | 764.05 |
| 2 | 2849.81 | 1779.65 | 1452.46 | 989.16 | 847.57 |
| 2 | 1866.26 | 1295.1 | 1175.19 | 891.7 | 781.08 |
| 2 | 2933.85 | 2823.94 | 2590.69 | 2385.6 | 2403.77 |
| 2 | 3322.39 | 2755.69 | 2259.22 | 2087.72 | 2241.13 |
| 2 | 2596.38 | 2542.32 | 2839.09 | 2240.81 | 2017.51 |
| 2 | 2496.42 | 2293.8 | 2259.27 | 1956.89 | 1803.65 |
| 2 | 2689.76 | 2613.5 | 2406.79 | 2207.88 | 2243.2 |
| 2 | 2667.24 | 1967.09 | 1535.46 | 1356.68 | 1083.1 |
| 2 | 2492.14 | 1912.53 | 1673.56 | 1406.59 | 1017.86 |
| 2 | 2711.65 | 2360.01 | 2149.44 | 1884.97 | 1738.11 |
| 2 | 3028.68 | 2720.65 | 1975.21 | 1477.99 | 1185.67 |
| 2 | 2191.72 | 1297.9 | 1022.75 | 935.29 | 1121.45 |
| 2 | 2199.6 | 2101.83 | 1948.22 | 1861.81 | 1747.8 |
| 2 | 1848.13 | 1423.33 | 1257.75 | 1061.01 | 902.35 |

Slopes data

| group | block1 | block2 | block3 | block4 | block5 |
| --- | --- | --- | --- | --- | --- |
| 1 | 221.29 | 195.89 | 136.23 | 75.08 | 49.51 |
| 1 | 289.2 | 197.91 | 124.89 | -19.88 | -55.3 |
| 1 | 280.14 | 83.27 | 23.6 | 53.18 | -118.4 |
| 1 | 85.65 | -6.02 | -25.46 | -46.61 | -27.68 |
| 1 | 246.73 | 158.05 | 57.83 | 67.54 | 195.05 |
| 1 | 281.26 | -22.7 | -204.69 | -93.24 | -154.05 |
| 1 | 450.24 | 136.95 | 250.23 | 487.05 | 396.83 |
| 1 | 347.57 | 372.49 | 516.25 | 426.78 | 367.97 |
| 1 | 253.28 | 307.83 | 250.25 | 184.81 | 243.45 |
| 1 | 279.29 | 345.17 | 349.95 | 296.4 | 189.74 |
| 1 | 291.43 | 189.29 | 38.45 | 98.26 | 56.75 |
| 1 | 205.31 | 128.88 | 81.63 | -36.38 | 15.56 |
| 1 | 161.64 | 134.82 | 69.34 | 97.46 | 3.93 |
| 1 | 157.58 | 172 | 24.65 | 68.34 | 198.03 |
| 1 | 305.4 | 145.85 | 136.21 | 213.33 | 233.34 |
| 1 | 231.55 | 264.26 | 160.44 | -40.25 | -181.17 |
| 1 | 277.19 | -36.55 | -102.64 | -58.57 | -57.8 |
| 2 | 118.48 | 40.87 | 40.26 | 66.44 | 42.03 |
| 2 | 138.55 | 145.6 | 133.91 | 189.52 | 163.94 |
| 2 | 171.64 | -12.82 | 14.14 | 20.14 | 35.57 |
| 2 | 184.75 | 18.44 | -57.7 | -91.21 | -52.99 |
| 2 | 232.93 | 105.04 | 52.97 | 15.82 | -10.33 |
| 2 | 410.48 | 223.84 | 142.09 | 243.85 | 125.86 |
| 2 | 448.28 | 288.07 | 126.27 | 97.06 | 83.25 |
| 2 | 181.98 | 44.83 | 85.74 | -61.55 | -39.56 |
| 2 | 169.61 | 198.47 | 123.19 | 76.22 | -49.88 |
| 2 | 294.9 | 318.99 | 211.55 | 216.94 | 231.69 |
| 2 | 207.44 | 123.15 | -147.3 | -89.44 | -84.59 |
| 2 | 80.4 | -100.32 | -183.06 | -136.62 | -30.53 |
| 2 | 313.28 | 363.38 | 95.4 | 42.99 | 30.03 |
| 2 | -63.09 | -145.2 | -452.16 | -336.97 | -204.09 |
| 2 | 112.6 | 22.49 | -53.25 | 65.86 | 9.17 |
| 2 | 273.04 | 221.31 | 162.31 | 178.53 | 188.37 |
| 2 | 169.24 | 87.05 | 66.23 | 16.48 | 30.72 |

Correlation data

| group | thought.use | slope100 |
| --- | --- | --- |
| 1 | 3 | 17 |
| 1 | 15 | 16 |
| 1 | 4 | 4 |
| 1 | 5 | 2 |
| 1 | 20 | 7 |
| 1 | 15 | 6 |
| 1 | 15 | 11 |
| 1 | 3 |  |
| 1 | 10 | 2 |
| 1 | 15 | 4 |
| 1 | 10 | 4 |
| 1 | 5 | 4 |
| 1 | 15 | 2 |
| 1 | 20 | 2 |
| 1 | 12 | 9 |
| 1 | 15 | 17 |
| 1 | 18 | 7 |
| 2 | 1 | 4 |
| 2 | 1 | 3 |
| 2 | 4 | 4 |
| 2 | 3 | 3 |
| 2 | 1 | 9 |
| 2 | 15 | 9 |
| 2 | 1 | 13 |
| 2 | 20 | 6 |
| 2 | 15 | 4 |
| 2 | 20 |  |
| 2 | 18 | 8 |
| 2 | 7 | 4 |
| 2 | 20 | 15 |
| 2 | 10 | 2 |
| 2 | 2 | 5 |
| 2 | 10 | 19 |
| 2 | 2 | 6 |
